# Immunologically relevant aspects of the new COVID-19 vaccines—an ÖGAI (Austrian Society for Allergology and Immunology) and AeDA (German Society for Applied Allergology) position paper

**DOI:** 10.1007/s40629-021-00178-2

**Published:** 2021-06-18

**Authors:** Eva Untersmayr, Elisabeth Förster-Waldl, Michael Bonelli, Kaan Boztug, Patrick M. Brunner, Thomas Eiwegger, Kathrin Eller, Lisa Göschl, Katharina Grabmeier-Pfistershammer, Wolfram Hötzenecker, Galateja Jordakieva, Alexander R. Moschen, Birgit Pfaller, Winfried Pickl, Walter Reinisch, Ursula Wiedermann, Ludger Klimek, Karl-Christian Bergmann, Randolf Brehler, Wolfgang Pfützner, Natalija Novak, Hans Merk, Uta Rabe, Wolfgang Schlenter, Johannes Ring, Wolfgang Wehrmann, Norbert Mülleneisen, Holger Wrede, Thomas Fuchs, Erika Jensen-Jarolim

**Affiliations:** 1 Institute of Pathophysiology and Allergy Research, Centre for Pathophysiology, Infectiology and Immunology, Medical University of Vienna, Waehringer Guertel 18–20, 1090 Vienna, Austria; 2 Department of Neonatology, Paediatric Intensive Care Medicine and Neuropaediatrics with Centre for Congenital Immunodeficiencies, University Clinics of Paediatrics and Adolescent Medicine, Medical University of Vienna, Vienna, Austria; 3 Clinical Department of Rheumatology, University Clinics of Internal Medicine III, Medical University of Vienna, Vienna, Austria; 4 Ludwig Boltzmann Institute for Rare and Undiagnosed Diseases, Vienna, Austria; 5 St. Anna Children’s Hospital and University Clinic for Paediatrics and Adolescent Medicine, Medical University of Vienna, Vienna, Austria; 6 St. Anna Kinderkrebsforschung GmbH, Vienna, Austria; 7 CeMM Research Centre for Molecular Medicine of the Austrian Academy of Sciences, Vienna, Austria; 8 University Clinics of Dermatology, Medical University of Vienna, Vienna, Austria; 9 Translational Medicine Program, Research Institute, Hospital for Sick Children, Toronto, Ontario Canada; 10 Department of Immunology, Faculty of Medicine, University of Toronto, Toronto, Ontario Canada; 11 Clinical Department of Pediatrics, University Hospital St. Pölten, St. Pölten, Austria; 12 Clinical Department of Nephrology, Internal Medicine, Medical University of Graz, Graz, Austria; 13 Institute of Immunology, Centre for Pathophysiology, Infectiology and Immunology, Medical University of Vienna, Vienna, Austria; 14 University Clinics of Dermatology and Venereology, Kepler University Hospital, Comprehensive Allergy Centre, Linz, Austria; 15 University Clinics of Physical Medicine, Rehabilitation and Occupational Medicine, Medical University of Vienna, Vienna, Austria; 16 University Clinics of Internal Medicine, Department of Gastroenterology and Hepatology, Kepler University Hospital, Linz, Austria; 17 Department of Internal Medicine 1, Karl Landsteiner University of Health Sciences, University Hospital, St. Pölten, Austria; 18 Clinical Division of Gastroenterology and Hepatology, University Clinics of Internal Medicine III, Medical University of Vienna, Vienna, Austria; 19 Institute of Specific Prophylaxis and Tropical Medicine, Centre for Pathophysiology, Infectiology and Immunology, Medical University of Vienna, Vienna, Austria; 20 Centre for Rhinology and Allergology, Wiesbaden, Germany; 21 Department of Dermatology, Venereology and Allergy, Charité—Universitätsmedizin Berlin, Berlin, Germany; 22 Department of Skin Diseases—General Dermatology and Venereology—Outpatient Clinic for Allergology, Occupational Dermatology and Environmental Medicine, University Hospital Münster, Münster, Germany; 23 Department of Dermatology and Allergology, University Hospital Marburg (UKGM), Philipps University Marburg, Marburg, Germany; 24 Clinic and Polyclinic for Dermatology and Allergology, University Hospital Bonn, Bonn, Germany; 25 Department of Dermatology and Allergology, RWTH Aachen University, Aachen, Germany; 26 Clinic for Allergology, Johanniter-Krankenhaus im Fläming Treuenbrietzen GmbH, Treuenbrietzen, Germany; 27 AeDA—Ärzteverband Deutscher Allergologen, Langen-Dreieich, Germany; 28 Skin and Laser Centre at the Opera, Munich, Germany; 29 Dermatological Group Practice Wehrmann, Münster, Germany; 30 Asthma and Allergy Centre Leverkusen, Leverkusen, Germany; 31 Nasal and Ear Specialist, Herford, Germany; 32 Department of Dermatology, Venereology and Allergology, University Medical Center Göttingen (UMG), Georg-August-University, Göttingen, Germany; 33 Interuniversity Messerli Research Institute, University of Veterinary Medicine Vienna, Medical University of Vienna, University of Vienna, Vienna, Austria

**Keywords:** COVID-19 vaccination, Immune response, Immunomodulation, Immunosuppression, Vaccination effect

## Abstract

**Background:**

The vaccines against the coronavirus disease 2019 (COVID-19) approved in the European Union represent a decisive step in the fight against the pandemic. The application of these available vaccines to patients with pre-existing immunological conditions leads to a multitude of questions regarding efficacy, side effects and the necessary patient information.

**Results:**

This review article provides insight into mechanisms of action of the currently available severe acute respiratory syndrome coronavirus 2 (SARS-CoV-2) vaccines and summarises the current state of science as well as expert recommendations regarding tolerability of the vaccines. In addition, the potential to develop protective immune responses is determined. A special focus is given on patients under immunosuppression or in treatment with immunomodulatory drugs. Special groups of the population such as children, pregnant women and the elderly are also considered.

**Conclusion:**

Despite the need for a patient-specific risk–benefit assessment, the consensus among experts is that patients with immunological diseases in particular benefit from the induced immune protection after COVID-19 vaccination and do not have an increased risk of side effects.

## General information on COVID-19 vaccinations

The novel severe acute respiratory syndrome coronavirus 2 (SARS-CoV-2), the trigger of COVID-19, has led to over 120 million ill patients and nearly 2.7 million deaths by the time of writing this review (16 March 2021). The primary interest of the global community is therefore to prevent further morbidity and mortality associated with COVID-19 [1]. To achieve this goal, effective vaccination against the novel virus is an essential strategy to maintain health services and public life while reducing social constraints [2]. In the context of COVID-19 vaccination, there are four vaccination objectives. Most important is (a) the prevention of severe COVID-19 courses and deaths. In addition, (b) the protection of persons with a particularly high, work-related risk of infection, (c) the prevention of disease transmission and (d) the maintenance of public life [3].

### Currently available COVID-19 vaccines and their principles of action

According to information from the World Health Organization (WHO), which is updated twice a week, a total of 81 COVID-19 vaccines are in clinical development and a further 182 are in a preclinical development phase as of 16 March 2021 (Table 1; [4]).

**Table 1:** COVID-19 vaccines in clinical development according to WHO^a^ (as of 16 March 2021) [4]

Vaccine manufacturing platforms	Number of vaccine candidates
Protein subunit	27
Viral vector (non-replicating)	12
DNA	11
Inactivated virus	10
RNA	10
Viral vector (replicating)	4
“Virus like particles”	3
VVr + antigen-presenting cell	2
Live-attenuated virus	1
VVnr + antigen presenting cell	1

^*a*^*WHO* World Health Organisation,* VVr* viral vector (replicating),* VVnr* viral vector (non-replicating)

Every vaccination against infectious diseases aims in principle to induce both humoral and cellular immunity against the vaccinated antigen(s) of the respective pathogen to ensure immediate recognition and protection by the immune system of the vaccinated person upon pathogen contact. Either the whole pathogen or parts of the virus, which are necessary for the invasion of the pathogens into body cells, can be used. If toxins are produced by the respective pathogens and are relevant for the disease, they can also be used for vaccination.

The mRNA-based SARS-CoV‑2 vaccines BNT162b2 (Corminaty®, Pfizer/BioNTech, Pfizer Inc, New York City, NY, US and Biontech SE, Mainz Germany) and mRNA-1273 (COVID-19 Vaccine Moderna ®, Moderna Inc. Cambridge, MA, US) break new ground regarding the delivery of the vaccine antigens. The mRNA-based vaccines do not deliver the antigen against which an immune response is mounted (surface protein of SARS-CoV-2), but the blueprint (the mRNA) for the production of the target protein. Muscle cells are the primary cells to take up the mRNA. Dendritic cells are also involved in the presentation of the antigen, with the advantage that they migrate to the regional lymph nodes and induce the immune response there. The target cells produce the viral antigen based on the information of the mRNA by rewriting it into the amino acid sequence of the protein structure. The generated protein becomes visible to the immune system of the vaccinated person as a surface protein on the cells. The immune system recognises the surface protein of the SARS-CoV‑2 as foreign and mounts an immune response. This consists of both specific antibodies and a specific cellular defence reaction. The decisive step is that degradation products of the proteins are presented to T‑cells in the form of peptides. These cells are strongly activated by these foreign peptides leading to an increased number of activated T cells and enables the T cells to recognise and kill virus-infected cells. Furthermore, activated T cells support the antibody-producing B cells by cytokine release.

As a result of the immune response, the body of the vaccinated person learns to defend itself against the infectious SARS-CoV‑2 pathogen even in the case of exposure and to prevent a COVID-19 illness or to reduce its severity.

Due to the rapid degradation of the mRNA, it has to be packed in a protective envelope to ensure uptake into the body’s cells (the transfection). This is accomplished with the help of liposomes. The composition of the mRNA-transporting and -protecting liposomes has been continuously improved in recent years, making it possible today to produce well working mRNA-based vaccines. Since both, the liposomes and the mRNA, can disintegrate very easily, these vaccines have to be stored at very low temperatures (−20 °C (mRNA-1273) to −70 °C (BNT162b2)) between production and use in humans, which involves considerable logistical effort and costly storage and distribution necessities.

The vaccine ChAdOx1‑S, synonymously AZD1222 (Vaxzevria®, AstraZeneca, Cambridge, UK), the vaccine Ad26.COV2.S (COVID-19 Vaccine Janssen, Johnson & Johnson, New Brunswick, NJ, US) and the vaccine Gam-COVID-Vac (rAd26‑S and rAd5‑S; rAd, recombinant adenovirus-based; known as Sputnik V, Gamaleja Institute, Moscow, Russia) are also new vaccine classes, so-called virus vector vaccines. They are based on harmless human or monkey viruses being no longer able to replicate, but can still infect cells. The information for the SARS-CoV‑2 surface protein is introduced into the susceptible body cells, which express the SARS-CoV‑2 surface protein on the cell surface leading to an activation of the immune system. The adenovirus-based vector vaccines can be stored in a refrigerator at 4 °C for several months without losing effectiveness.

One general disadvantage of virus vector vaccines is that they cannot be used to vaccinate several times, as the vaccinee forms neutralising antibodies against the vector itself. This means that booster vaccinations are reduced or not at all effective. This is not a problem with the vaccine Ad26COV2.S, which only has to be administered once. If booster vaccinations are necessary, an alternative strategy is applied using for the initial immunisation vector virus type A and for a booster the heterologous vector vaccine type B. This has already become a reality in the randomized, placebo-controlled study with Gam-COVID-Vac (rAd26‑S and rAd5-S) [5].

A traditional technology in the production of vaccines is the use of inactivated pathogens. For the production of these vaccines, the pathogens are inactivated by chemical or physical action, ensuring that they cannot replicate or can only replicate to a limited extent and are no longer infectious for immunocompetent persons. There are currently a total of 10 vaccine candidates against SARS-CoV‑2 based on this technology. Among them is CoronaVac (Sinovac Biotech, Beijing, China, the vaccine that received emergency approval in China in summer 2020. In phase II studies having now been published, the vaccine shows good efficacy and good tolerability [6, 7]. Further phase III studies have not yet been published.

The subunit vaccination technology, which has already been widely and very successfully used for a long time in the context of vaccinations against other dangerous infectious diseases (such as hepatitis B and influenza), is used to produce the SARS-CoV‑2 vaccine “full-length recombinant SARS-CoV‑2 glycoprotein nanoparticle vaccine adjuvanted with Matrix M” (synonym NVX-CoV2373; Novavax, Gaithersburg, MA, US). The SARS-CoV‑2 surface protein, which is important for SARS-CoV‑2 infection, is produced recombinantly, purified and, and inoculated mixed with appropriate adjuvants. In subunit vaccines completely harmless antigens are injected into the body. In the body, the protein is recognised as foreign by immune cells, triggering the immune response. The advantage of subunit vaccines is that a substance defined on a molecular basis is used with the actual foreign antigen. Subunit vaccines can be vaccinated repeatedly without limiting vaccination effect (see hepatitis B vaccines), but usually require adjuvants as active ingredient boosters.

Vaccinations are, thus, intended to teach the immune system to prevent the disease or, ideally, the infection. In addition to safety, the immunogenicity and efficacy are also tested in the approval studies.

### Information on vaccination schedules

Most vaccinations require multiple doses in the course of primary immunisations, either to achieve the highest possible level of immunity or to allow individuals who have not responded sufficiently to a single dose to renew the immune response. After primary immunization, the number of possible additional doses of a vaccination series can be influenced by certain risk factors such as age or immunosuppression. According to current knowledge and approval, the COVID-19 vaccines approved to date require two partial vaccinations in order to achieve the most pronounced immunity possible, although the duration of the vaccine protection is currently not known. With regard to further booster vaccinations, there are currently no concrete recommendations for the approved vaccines. The duration of immunogenicity and protection is currently being tested by the manufacturers in ongoing studies.

The recommended interval between the two initial vaccinations with the BNT162b2 vaccine is 21 days (19–42 days). After only one injection, protection against the SARS-CoV‑2 virus can be expected in 52.4% of cases (short-term protection starts around day 10), increasing to 95% after the 2nd vaccination [8]. For the mRNA-1273 vaccine, the recommended interval is 28 days (21–42 days), with a protection rate of 80.2% after the 1st injection and 94% after the 2nd injection [9]. In animal experiments, a sharp drop in virus-neutralising antibodies was observed approximately 28 days after the first injection of mRNA vaccinations against SARS-CoV‑2. An increase in antibodies could be strongly stimulated by a 2nd vaccination [10]. An extension of the interval between the two BNT162b2 vaccine doses for up to 42 days (6 weeks) is generally considered permissible for WHO in the case of supply shortage [11]. In order to ensure maximum effectiveness, it is nevertheless recommended to adhere as closely as possible to the vaccination schedule recommended by the manufacturer.

According to the latest results, the vector-based vaccine AZD1222 has an efficacy of 94% after the 1st vaccination (prevention of hospitalisation) [12]. The registration data revealed a 64.1% efficacy after only 1 injection and 70.4% after the 2nd vaccination [13]. Better vaccination results were observed with longer intervals, which is the reason for the 11 to 12 weeks interval between the two vaccinations currently recommended [14, 15].

The vaccine Ad26COV2.S, which was approved in the EU on 11 March and can be stored at normal refrigerator temperatures, shows a good protective effect in more than 90% of the study participants already after a single dose according to first published data [16]. According to press releases from the manufacturer, there was also a good protective effect against COVID-19 in the phase 3 study (ENSEMBLE) [17]. In South Africa, however, where the SARS-CoV‑2 variant B.1.351 dominated, the protective effect was reduced. Publication of these data in a scientific journal is still pending.

Due to the current lack of data from clinical trials, it is not recommended to combine different vaccines. Based on promising murine data, it is currently being tested to perform booster vaccinations with a different vaccine after the first vaccination with one vaccine. It remains to be seen whether improved clinical efficacy can be achieved.

## The COVID-19 vaccination in everyday clinical practice

### In patients with a history of COVID-19 disease

Due to the different reactivity patterns in individual patients, a general conclusion on the time period of protection against a re-infection after a COVID-19 infection is currently not possible. Initial studies predicted sustained immune protection after infection, based on the presence of memory B cells producing virus-specific antibody subclasses other than IgM [18]. Furthermore, the presence of antigen-specific B and T cells 6 to 8 months after infection is considered as indicative of an existing virus-specific immune response [19–24]. This is in contrast to other studies reporting a rapid decrease in IgA antibody titers after natural infection [25]. The protective effect of the antibodies produced during a natural infection seems to correlate with the amount of antibodies (antibody titer in the blood). In turn, the extent of antibody production correlates with the severity of the disease [26, 27]. It is recommended to get vaccinated even after having been infected with COVID-19. Current studies do not indicate that vaccination is problematic or associated with more side effects after a previous infection. According to the recommendations of the Austrian National Vaccination Committee, an interval of 6 to 8 months is recommended between a previous SARS-CoV‑2 infection and the COVID-19 vaccinations. Due to shortage of vaccine, those persons, who do not yet have an immunity, should be vaccinated first [28]. If a SARS-CoV‑2 infection occurs after administration of the first vaccine dose, the Permanent Vaccination Commission of the Robert Koch Institute and the Austrian National Vaccination Committee currently recommend that the second injection be administered six months after recovery or diagnosis [3, 28]. Vaccination is not recommended in patients suffering from active symptoms of COVID-19 disease or being tested positive for SARS-CoV‑2. However, it is not recommended to test for COVID-19 antibodies prior to vaccination, as these tests currently do not provide sufficient information regarding immunity.

### In context of allergy and autoimmunity

The risk for IgE-mediated severe vaccine side effects due to anaphylaxis as well as precautions measures and anaphylaxis management are discussed in the mini-review “Answers to burning questions for clinical allergologists related to the new COVID-19 vaccines”. In case of an allergic/anaphylactic reaction after the administration of the COVID-19 vaccine as well as for a small population at higher risk for an anaphylactic reaction, an allergological work-up is recommended before the first vaccination [29–31].

Large studies have reported the development of autoantibodies after COVID-19, which were also shown to be associated with disease severity [32–34]. The observed neurological impairment in COVID-19 [35] has been linked to both a direct effect of SARS-CoV‑2 on neurons and to autoimmune mechanisms [36]. A higher number of peripheral neuropathies (Guillain–Barré syndrome) and polyneuropathies with associated muscle denervation are observed in SARS-CoV-2 patients [37], which could be part of Long-COVID [34, 38]. Autoimmune haematological phenomena such as thrombocytopenic purpura or autoimmune haemolytic anaemia were also observed in SARS-CoV-2 patients [39]. A Danish population study showed a more severe course in patients with pre-existing autoimmune diseases, especially under systemic steroid therapy [40]. In a study with monoclonal antibodies against the spike protein, a reaction with 55 different tissues was reported due to epitope similarities of the SARS-CoV‑2 protein with mitochondrial antigen M2, F‑actin and thyroid peroxidase. Furthermore, cross-reactivity with retinal pigment epithelial surface transport proteins has been predicted, currently without clinical evidence, which should be further monitored with regard to autoimmune retinopathy [41]. While initially women in particular were concerned about reduced fertility after SARS-CoV‑2 [42], which has also led to vaccination anxiety, there are now initial reports that male fertility might be impaired after SARS-CoV‑2 infection due to autoimmunity and impaired spermatogenesis [43]. This is due to reports of SARS-CoV‑2 receptor expression on tissues of the female and male genital tract [44]. However, there is no evidence that there is a risk in connection with the vaccinations, which is why vaccination is clearly recommended, especially in those who wish to have children [28, 45].

Although concerns have also been raised about COVID-19 vaccination in the general population due to these observed autoimmunity reactions associated with SARS-CoV‑2 disease, it has be clearly stated that there is no evidence to date that the vaccines might lead to autoimmune reactions.

In childhood, a hyperinflammatory syndrome (PIMS-TS, “paediatric inflammatory multisystem syndrome temporally related to SARS-CoV-2”; MIS‑C, “multisystem inflammatory syndrome in children”) has been observed in the context of SARS-CoV‑2 infections, which clinically resembles the Kawasaki syndrome [46]. Clinical trials for SARS-CoV‑2 vaccination in children and adolescents will therefore pay special attention to related side effects.

### In patients on immunomodulatory drugs

COVID-19 vaccination is also generally recommended for patients on immunosuppressive and immunomodulatory therapy, regardless of their mode of action [28]. This includes, among others, patients with autoimmune diseases and after transplantation [47]. In general, live vaccines should not be used in these patients [48, 49]. The currently licensed SARS-CoV‑2 vaccines are not live vaccines. From the current perspective, it is unclear whether patients with autoimmune diseases under therapies show differences in tolerability and/or efficacy between an mRNA- and a vector-based vaccine. As of 23 February 2021, the Austrian National Vaccination Committee recommends the use of a mRNA-based vaccine in high-risk patients, including patients on immunosuppressive therapy and biologics, if available [28]. Recommendations on optimised timing of COVID-19 vaccination schedule in relation to the cyclic administration of a biologic cannot be made at this time. As with other inactivated vaccines, vaccination should be done according to available recommendations and the national vaccination schedule [30, 48, 49]. It should be noted, however, that certain active substances and classes, such as systemic glucocorticoids, thiopurines, methotrexate, TNF or JAK inhibitors can generally lead to a reduced vaccination response. In view of the potentially reduced vaccination response under these therapies, the vaccination response should be investigated and persons living in the same household should also be vaccinated as a prophylaxis measure.

Patients with ongoing B‑cell depleting therapy (rituximab, obinutuzumab and others) are in a special situation, as they are likely to have a significantly increased risk of a severe course of COVID-19 disease [50]. In addition, it is unclear whether a robust vaccine response can be achieved in these patients. It is currently assumed that people with a B-cell percentage of less than 2% will not benefit from vaccination and therefore vaccination prophylaxis of social contacts is of particular importance. In this patient population, it will be particularly important to choose the best possible timing for vaccination and to select the most potent vaccine to achieve a vaccine response. Again, vaccination response should be checked in these patients.

Biomarkers for the efficiency of mRNA-based COVID-19 vaccination under immunosuppressive and immunomodulating therapy would be of great clinical relevance. Whether seroconversion will be useful remains to be proven. Neutralising antibody titers or receptor-binding domain-specific antibodies can be determined. Alternatively, commercially available SARS-CoV‑2 test systems detecting the T‑cell response could be used. In view of the still unclear situation regarding a possible sterile immunity under mRNA-based COVID-19 vaccination, a possible “virus shedding” and its duration under immunosuppressive and modulating therapy should also be precisely ascertained in the case of SARS-CoV‑2 infection after vaccination [51]. In any case, more studies are needed to examine prolonged virus shedding after SARS-CoV‑2 infection of vaccinated patients under concomitant immunomodulating therapies.

The administration of plasma from convalescent COVID-19 patients, which contains neutralising natural antibodies, could represent a treatment option for patients with reduced antibody production, which should be examined [52]. When using this therapeutic approach, in addition to the timing of the start of therapy, precise characterisation of the donor sera for specificity, concentration and function of SARS-CoV‑2 specific antibodies prior to application is advantageous. This would also explain study results, which reported no significant advantage of this therapeutic approach [53].

### In the context of organ and bone marrow transplantation

Post-transplant patients have a significantly higher risk of becoming severely ill with SARS-CoV‑2 infection, requiring mechanical ventilation, and of dying from COVID-19 [54]. According to a recent publication, the risk of dying from COVID-19 in the first year after kidney transplantation is higher than remaining on dialysis [55]. Therefore, patients after organ or bone marrow transplantation are to be classified as high-risk patients [56]. Thus, there is urgent indication to vaccinate this population. So far, the mRNA-based vaccines have shown a robust vaccine response in the normal population during the currently available follow-up period. It is still unclear how effective the vaccines will be in patients who have undergone organ or bone marrow transplantation. Due to the severe immunocompromise in the early phase after transplantation, most transplant centres recommend that their patients receive vaccination not earlier than two months after transplantation [48]. In exceptional cases—such as influenza vaccination—immunisation is also considered as early as one month after solid organ transplantation (depending on the influenza season) [48]. For patients after stem cell transplantation, immunisation is recommended at the earliest six months after transplantation [48]. In all patients after transplantation, however, the success of the vaccination should be checked by determining either neutralising antibodies or receptor binding domain (RBD)/S1-specific antibodies one month after vaccination. In addition, Austrotransplant, the Austrian Society for Transplantation, Transfusion and Genetics, currently recommends the use of mRNA-based vaccines in patients after transplantation, as a more robust vaccination response can be expected compared to the currently available vector vaccines. However, it should be emphasised that patients on the transplant waiting list should be vaccinated before transplantation. Since the vaccination success can also be impaired in these patients, it will also make sense to monitor the vaccination response. In addition, relatives living in the close vicinity to the transplanted patient should also be vaccinated in order to provide the patient with the best possible protection against infection.

### In congenital or iatrogenic coagulation disorders

Patients with congenital coagulation disorders are not considered to be at risk for severe COVID-19. The scientific societies currently do not recommend any restriction with regard to vaccination. Consultation with the treating haematologist is only recommended in the case of von Willebrand syndrome and rare coagulation disorders [57]. Studies suggest that there is no increased risk of complications following intramuscular vaccination when patients are receiving oral anticoagulation therapy. Similarly, no benefit has been shown with subcutaneous injection in this patient group [58]. It is explicitly advised against subcutaneous administration of mRNA vaccines. Scientific societies recommend checking the International Normalised Ratio (INR) 72 h before intramuscular injection [57]. In the context of COVID-19 vaccines, oral and intranasal routes of administration are under development, but the corresponding vaccines are currently in phase 1 or phase 1/2 of clinical testing [4]. Preclinical data suggest that when administered intranasally, a single dose of the licensed vaccine AZD1222 protects against SARS-CoV‑2 infection and transmission [59].

By March 16, 2021, after approximately 20 million vaccinations with AZD1222 in Europe, thromboembolic events have been reported to the European Medicines Agency (EMA) in 25 patients in temporal context to the intramuscular injection [60]. In a group of 9 patients (8 female patients and 1 male patient), the clinical symptoms were evaluated in detail and a similarity to heparin-induced thrombocytopenia was found. In a subgroup of patients, antibodies against platelet factor 4 were detected, which induced platelet activation [61]. Based on these data, recommendations for the diagnosis and therapy of “virus/vaccine induced prothrombotic immune thrombocytopenia” (VIPIT) were published [62, 63].

## Recommendations regarding COVID-19 vaccination for special groups of the population

### In children and adolescents

The vaccination of children and adolescents has to be regarded as a priority goal in order to sustainably reduce infection rates and to be able to sustainably reduce currently necessary hygiene measures. Since the outbreak of the pandemic, this cohort has often been regarded as a facilitator for infection, even if available data do not necessarily support this [64]. For other respiratory infectious diseases, it has been proven that vaccination of children and adolescents has clear epidemiological advantages for the entire population [65]. However, the dose, tolerability and efficacy in children under 16 years of age are currently still completely unclear.

Currently, registration trials for SARS-CoV‑2 vaccination for children and adolescents up to 16 years of age have not been completed and therefore SARS-CoV‑2 vaccination cannot be recommended in this age group at present. In the EU, the mRNA vaccine BNT162b2 is currently licensed from 16 years of age, the mRNA vaccine mRNA-1273 from 18 years of age, the vector vaccine AZD1222 and the vector vaccine Ad26.COV2.S from 18 years of age.

The following clinical trials are currently ongoing for children and adolescents: The mRNA vaccine BNT162b2 has been tested in subjects aged 12 to 16 years in the adult dose with two vaccine doses three weeks apart since October 2020. In the USA, this vaccine will be tested in children between 5 and 11 years of age in the coming months. An additional trial in children under 5 years of age is planned for the end of 2021. The mRNA vaccine mRNA-1273 has been tested in 3000 children between 12 and 17 years of age in the adult dose in the “TeenCove study” since December 2020. The AZD1222 vaccine will be tested in 6‑ to 18-year-olds. For the Ad26.COV2.S, phase 2 trials in children over 12 years of age have been ongoing since August 2020. Furthermore, a trial with the inactivated SARS-CoV‑2 vaccine BBIBP-CorV (Sinopharm COVID-19 vaccine) is ongoing for children aged three years and older.

### In pregnant women

Due to the lack of prospective data for the COVID-19 vaccine, there is currently no general vaccination recommendation for pregnant women. The recommendations for use of the Austrian National Vaccination Committee clearly refer to a risk–benefit assessment in pregnant women [28]. The German Society of Gynaecology and Obstetrics (DGGG), together with the working group Obstetrics and Prenatal Medicine (AGG) in the DGGG, the BLFG, the DGGEF, DGPGM, DGPM, DGRM, the URZ, the DVR and the BVF has written a statement emphasising that pregnant women should not be excluded from vaccination programmes [66]. Together with the attending physician, a decision on the use of mRNA-COVID-19 vaccination should be made with the pregnant woman after weighing the individual advantages and potential side effects. This joint decision should be preceded by a detailed information to allow conclusions on exposure risks, pre-existing comorbidities and individual and pregnancy-specific risk of SARS-CoV‑2 infection to be included into the decision-making process. The American College of Obstetricians and Gynecologists and also the Society for Maternal Fetal Medicine (SMFM) recommend that pregnant women should be given access to the mRNA vaccines after detailed information [67]. With regard to the decision to administer the AZD1222 vaccine, the EMA recommends a detailed medical discussion to weigh the potential risks and benefits for mother and child [66, 68]. In order to generate prospective safety data, a trial of the BNT162b2 vaccine has been initiated, enrolling a total of 4000 pregnant women to evaluate the efficacy and safety of the mRNA vaccine in pregnancy. Vaccination is recommended for women planning to have children (pre-conceptional).

### In older persons in the context of immunosenescence and the increased risk of severe COVID-19 disease

Immunosenescence is the term used to describe the declining function of the innate and adaptive immune system, which is associated with an increase in morbidity and mortality due to infectious diseases, including those related to viral infections [69]. Due to the continuous reduction of the thymus from sexual maturity, the number of naïve T cells decreases during life; effector and memory T cells dominate [70]. The resulting change with regard to the cytokine environment also has an effect on the maturation of B cells and antibody production. In addition, IgG^+^IgD^−^CD27^−^ double-negative B cells accumulate with older age, which is interpreted as exhaustion due to continuous stimulation or altered formation of the germinal centres [71]. It is now well documented that the risk of a more severe COVID-19 course and the number of hospitalised patients and deaths associated with SARS-CoV‑2 infection increases significantly from the age of 65 [72, 73]. Vaccination of vulnerable persons of older age is therefore a high priority in the national vaccination strategy [14, 28].

### In primary and acquired immunodeficiencies

Recent data show that at least a proportion of patients with primary immunodeficiency—especially those in whom the type I interphone response is limited by genetic defect or autoantibodies—are at increased risk for severe COVID-19 courses [32, 74, 75]. The efficacy of COVID-19 vaccine for people with immunodeficiencies has not really been studied as, with the exception of people with well-controlled HIV infection (on therapy and with a CD4^+^ T‑cell count > 500), none of the registration trials included immunosuppressed subjects. As the data on the HIV-positive study participants have not been published separately, no conclusions can be drawn. For an efficient immune response, an intact immune system is needed, with functioning antigen presentation as well as B‑cell and T‑cell responses. If parts are missing or weak, there may be a poorer vaccine response associated with lower protection in congenital or acquired defects in both B and T cells, as well as patients after splenectomy, immunosuppressive therapy directed against B or T cells, but also chemotherapy or high-dose steroid therapy. Nevertheless, many scientific societies (e.g. ESID, European Society for Immunodeficiencies; British Society of Immunology; BHIVA, British HIV Association; UN-AIDS, United Nations Programme on HIV/AIDS; CDC, Centers for Disease Control and Prevention) recommend COVID-19 vaccination for patients with immunodeficiencies. This is based on experience for other inactivated vaccines showing that a protective effect can be achieved at least partially (depending on the type and severity of the immunodeficiency). Data exist for influenza, HPV and herpes zoster vaccines [76–80]. It should be noted, however, that in the case of most vaccinations that have been investigated so far, it can be assumed that at least some of the patients (especially those with acquired immunodeficiencies or those that first manifest themselves in adulthood) already have an immunological memory for the vaccine antigen (e.g. through childhood vaccination). This means that the vaccination “only” needs to trigger an amplification/refreshment of the immune response, whereas SARS-CoV‑2 represents a neo-antigen for everyone, which could have consequences for the effectiveness of the vaccination.

Even in the complete absence of a B-cell response, vaccination is recommended by some scientific societies, since (T-) cellular immune responses also contribute to a protective effect. For COVID-19 in particular, there are increasing data that the cellular immune memory is important for protection against reinfection after a previous infection [81, 82]. However, this might not reflect the situation after vaccination with the viral spike antigens. Therefore, vaccination in the absence of B cells is not supported by the Austrian National Vaccination Committee.

With regard to the safety of previously established inactivated vaccines, there is no evidence for more severe or specific vaccine side effects in patients with immunodeficiencies compared to the normal population [83]. There are no data for mRNA vaccines, which have only been used sporadically in the context of oncological therapies. As a theoretical consideration, a possibly enhanced interferon‑I response to mRNA vaccines compared to other vaccine types might be found in patients with immunodeficiencies associated with autoimmune manifestations or iatrogenically caused by the therapy of an autoimmune disease [84], which could lead to an aggravation of autoimmune reactions. In terms of risk assessment, COVID-19 itself also carries a significant risk of aggravating autoimmune diseases [32]. With live vaccines (vaccinations with attenuated pathogens), there is a possibility that the vaccination could trigger the disease in patients with immunosuppression. Live vaccines are therefore not recommended for patients with immunodeficiencies. There are exceptions depending on the exact immunodeficiency and the severity of the immunodeficiency. However, no live COVID-19 vaccine is currently licensed or in the process of being licensed in the European Union.

There are currently no recommendations for monitoring vaccination response, nor are there any recommendations for a different dosage or additional booster doses in patients with immunodeficiencies [85].

## Documentation, efficacy and reactions upon vaccination

The following minimum requirements for vaccination documentation are mandatory in Austria: name of the vaccinating physician, the vaccinated person and the batch of the vaccine. In Austria, vaccination documentation is done via the newly established e‑vaccination system, which covers the documentation obligation of vaccination and in which all vaccinations are stored for lifetime. For the time being, no reduction of social restrictions after vaccination is planned, as it cannot yet be estimated whether all vaccination goals will be achieved through vaccination and whether a blockage of transmission will also be achieved. However, it can already be assumed that after vaccination with the vaccines currently available on the market, the vaccinated persons are less infectious due to a lower viral load [86, 87]. Following an interim evaluation of the use of mRNA vaccination in the USA, the CDC has changed its recommendations that fully immunised persons no longer have to be quarantined after contact with a COVID-19 patient [88].

Immunity after vaccination with the vaccine classes described above differs from (possible) immunity after SARS-CoV‑2 infection. The main reason for this is the “vaccine antigen”, which for all vaccines listed above is the SARS-CoV‑2 surface protein. This selective contact of vaccinated persons with the SARS-CoV‑2 surface protein distinguishes them from persons after having undergone SARS-CoV‑2 infection, who form antibodies against a variety of other virus components, such as matrix proteins, nucleocapsid proteins, envelope proteins or non-structural SARS-CoV‑2 proteins. This will also make it possible to distinguish an infection (antibodies against nucleocapsid proteins and surface proteins) from a vaccination with the above-mentioned vaccines (antibodies only against surface proteins). However, in order to be able to assess immunity, highly specific tests (for antibodies and cellular tests) will be needed that can easily determine the virus neutralisation potential of the induced antibodies and the T cells or killer cells respectively (surrogate neutralisation tests).

The immunogenicity goals consist of the formation and serological detection (seroconversion) of neutralising antibody titers, namely spike antigen-specific, neutralising antibodies, although it is currently not clear at what antibody titer is associated with protection. The desired efficacy of vaccination manifests itself in the prevention of a symptomatic COVID-19 disease. Sufficient immunity is expected approximately seven days after the 2nd injection with the vaccine BNT162b2 and approximately after 14 days with mRNA-1273. The vaccine AZD1222 should already show a protective effect three weeks after the 1st vaccine shot; the 2nd vaccination should consolidate the vaccination success [89].

The extent of protection after SARS-CoV‑2 infection as well as during vaccination correlates positively with the level of neutralising immunoglobulins produced and is additionally dependent on mechanisms of the natural immune response (“trained immunity”) such as type I interferons or mucosal immunity [90].

Sterilising immunity is the term used to describe the neutralising antibodies that protect against (re)infection after a previous infection or vaccination. Whether the vaccinated person is still infectious for others after one of the currently approved COVID-19 vaccinations, i.e. whether there is protection against transmission, cannot be answered with certainty at this point in time.

Due to the induced immune response, the COVID-19 vaccines cause vaccination reactions, which are to be expected as harmless complaints after vaccinations. A distinction is made here between local reactions, such as burning, pain, hardening and reddening at the injection site, and systemic reactions, such as headache, fatigue, malaise, fever, chills, arthralgia, myalgia, nausea, vomiting and diarrhoea. After vaccinations with mRNA vaccines, these vaccination reactions are observed very frequently (in more than 1 in 10 patients) and occur mainly after the 2nd vaccination. With the vector vaccine AZD1222, the vaccination reactions occur more frequently after the first vaccination. It is important to clearly indicate during patient information that vaccination reactions are to be expected. Prophylactic administration of paracetamol (taking into account general contraindications) about six hours after vaccination can attenuate the vaccination reactions. If necessary, the medication can be continued every six hours for 24 to 48 h. Vaccine adverse reactions, i.e. reactions to vaccination that are not expected and go beyond the usual extent of a vaccination reaction, are reported nationally and are continuously assessed by the competent authorities. In early March 2021, due to the temporal context between vaccinations with AZD1222 and thromboembolic events, the use of AZD1222 was temporarily discontinued in several European countries. Even after a possible causal relationship between rare thromboembolic events and vaccinations with AZD1222 was established, the EMA again issued a positive benefit–risk assessment on 7 April 2021 and recommended the continuation of vaccinations [91]. Based on this, the national vaccination committees have published guidelines for vaccination with AZD1222, which are continuously reviewed and adapted according to the available data.

## Conclusion

Based on the available data on the SARS-CoV‑2 vaccines in adult patients with immunological diseases summarised here, it can be stated that after a precise weighing of the benefit–risk profile of the available vaccines, the recommendation clearly speaks in favour of the COVID-19 vaccination. Patient group-specific as well as age group-specific recommendations are to be implemented according to the recommendations for use of the national vaccination committees [28]. Due to the vaccine shortage, there are clear prioritisations for vaccination that precisely define high-risk patients as well as patients under immunosuppressive therapy [56]. Although the protective effect of vaccination cannot yet be assessed in detail, especially for patients on immunomodulatory or immunosuppressive therapy, a benefit for patients with immunological diseases can be expected. It will be essential to generate evidence through academic studies and accurate clinical documentation that will allow sufficient information regarding the safety and efficacy of the available COVID-19 vaccines for patients under immunomodulatory and immunosuppressive therapy or with immunodeficiencies.

## Abbreviations

AGG: Working Group Obstetrics and Prenatal Medicine in the DGGG
BHIVA: British HIV Association
BLFG: Bundesarbeitsgemeinschaft Leitender Ärztinnen und Ärzte in der Frauenheilkunde und Geburtshilfe (Federal Association of Senior Physicians in Gynaecology and Obstetrics)
BVF: Professional Association of Gynaecologists
CDC: Centers for Disease Control and Prevention
DGGEF: Deutsche Gesellschaft für Gynäkologische Endokrinologie und Fortpflanzungsmedizin (German Society for Gynaecological Endocrinology and Reproductive Medicine)
DGGG: German Society of Gynaecology and Obstetrics
DGPGM: German Society for Prenatal and Obstetric Medicine
DGPM: German Society for Perinatal Medicine
DGRM: German Society for Reproductive Medicine
DVR: Association for Reproductive Biology and Medicine
EMA: European Medicines Agency
ESID: European Society for Immunodeficiencies
INR: “International Normalized Ratio” (laboratory parameter indicating the functional performance of the extrinsic system of blood clotting)
JAK inhibitor: Janus kinase inhibitor
MIS‑C: Multisystem inflammatory syndrome in children
MRNA: Messenger RNA
PIMS-TS: Paediatric inflammatory multisystem syndrome temporally related to SARS-CoV‑2
SARS-CoV‑2: Severe acute respiratory syndrome coronavirus 2
TNF inhibitor: Tumour necrosis factor inhibitor
UN-AIDS: United Nations Programme on HIV/AIDS
URZ: Working Group of University Reproductive Medicine Centres of the DGGG
VIPIT: Virus/vaccine induced prothrombotic immune thrombocytopenia
WHO: World Health Organization
